# The hidden side of body integrity dysphoria: aberrant limbic responses to dynamic touch

**DOI:** 10.1093/braincomms/fcaf209

**Published:** 2025-05-28

**Authors:** Laura Zapparoli, Eraldo Paulesu, Martina Gandola, Gerardo Salvato, Gianluca Saetta, Marika Mariano, Francantonio Devoto, Silvia Amaryllis Claudia Squarza, Mariangela Piano, Peter Brugger, Gabriella Bottini

**Affiliations:** Psychology Department and NeuroMi—Milan Centre for Neuroscience, University of Milano-Bicocca, Milan 20126, Italy; fMRI Unit, IRCCS Orthopedic Institute Galeazzi, Milan 20151, Italy; Psychology Department and NeuroMi—Milan Centre for Neuroscience, University of Milano-Bicocca, Milan 20126, Italy; fMRI Unit, IRCCS Orthopedic Institute Galeazzi, Milan 20151, Italy; Department of Brain and Behavioral Sciences, University of Pavia, Pavia 27100, Italy; Cognitive Neuropsychology Centre, ASST Grande Ospedale Metropolitano Niguarda, Milan 20162, Italy; NeuroMi, Milan Center for Neuroscience, Milan 20126, Italy; Department of Brain and Behavioral Sciences, University of Pavia, Pavia 27100, Italy; Cognitive Neuropsychology Centre, ASST Grande Ospedale Metropolitano Niguarda, Milan 20162, Italy; NeuroMi, Milan Center for Neuroscience, Milan 20126, Italy; Professorship for Social Brain Sciences, Department of Humanities, Social and Political Sciences, ETH Zurich, Zurich 8092, Switzerland; Department of Adult Psychiatry, Psychiatry St. Gallen, Pfäfers 7312, Switzerland; Psychology Department and NeuroMi—Milan Centre for Neuroscience, University of Milano-Bicocca, Milan 20126, Italy; Psychology Department and NeuroMi—Milan Centre for Neuroscience, University of Milano-Bicocca, Milan 20126, Italy; Neuroradiology Department, ASST Grande Ospedale Metropolitano Niguarda, Milan 20162, Italy; Neuroradiology Department, ASST Grande Ospedale Metropolitano Niguarda, Milan 20162, Italy; Center for Psychiatric Research, Adult Psychiatry and Psychotherapy, University Hospital Zurich, Zurich 8006, Switzerland; Department of Brain and Behavioral Sciences, University of Pavia, Pavia 27100, Italy; Cognitive Neuropsychology Centre, ASST Grande Ospedale Metropolitano Niguarda, Milan 20162, Italy; NeuroMi, Milan Center for Neuroscience, Milan 20126, Italy

**Keywords:** body integrity dysphoria, xenomelia, body ownership, fMRI, tactile stimulation

## Abstract

Body integrity dysphoria (BID) is a brain-based disorder characterized by a persistent, obsessing and disturbing desire for the amputation of healthy limbs. Importantly, individuals with BID are adamant about which specific body segment they wish to have amputated and the exact level at which the amputation should occur. The condition has been linked to altered resting-state brain functional connectivity and task-based activity at the level of somatosensory cortices. However, the inevitable distress associated with the condition has not been explained by current neurophysiological data. In this functional MRI (fMRI) study, we studied individuals with a lifelong desire for the amputation of their left leg using a dynamic somatosensory stimulation paradigm. We identified and marked the desired line of amputation on the BID individuals’ left leg and a corresponding point on their right leg. We measured brain activations in response to stimulation of the lower limbs while participants were instructed to focus on the tactile sensations and detect when the stimulation crossed the line of the desired amputation. Compared with healthy controls focusing on the same segments of their legs, individuals with BID showed higher neural activations specifically for the stimulation of their left leg in a large cortical and subcortical neural network primarily associated with rewarding and pain stimuli. Some of these hyperactivations were particularly marked immediately after the stimulation had passed over the line of the desired amputation. When the stimulation crossed the desired point of amputation, there were also increased activations in the premotor cortices and the anterior cingulum, a sign of premotor attentional arousal. Our data show a pathological relationship between altered neural representations of the body map and the brain reward system, connecting BID to the visceral brain for the first time.

## Teaser

Zapparoli *et al*. reported that individuals with body integrity dysphoria (BID) show heightened limbic and reward-related brain activations in response to tactile stimulation of the affected limb. These findings suggest that BID involves altered somatosensory and affective processing, linking body representation disturbances to the brain’s emotional and reward systems.

## Graphical Abstract

**Graphical Abstract:**
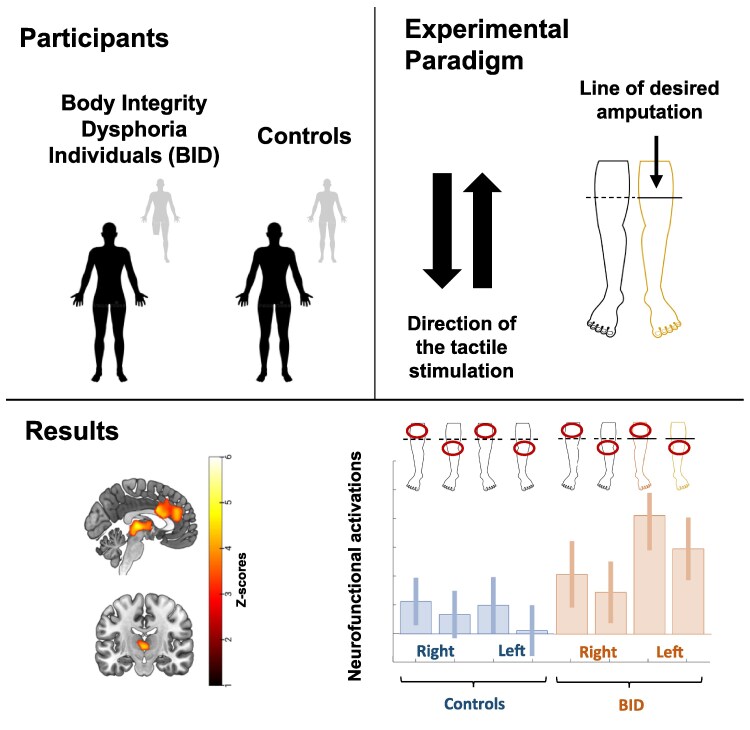

## Introduction

Body integrity dysphoria (BID) is characterized by a persistent and intense desire to have one, or even more, of their physically healthy limbs amputated. This overwhelming need often drives individuals with BID to seek surgical amputation and, in more extreme scenarios, to attempt amputation on their own. A variation of this condition also manifests as a desire for limbs to become non-functional (paralysed), further complicating the clinical understanding and indicating a multifaceted nature of BID. The present contribution focuses, however, on the amputation variant of BID.

The onset of the disease typically begins in early childhood, and individuals with BID often show paraphilic interests (i.e. erotic attraction towards paraplegic and amputee bodies and/or sexual arousal following the idea of being amputated; see also^1,2^). They also often engage in pretending behaviours like mimicking the status of an amputee by moving in a wheelchair, using crutches or binding up the affected limb: this can result in harmful consequences such as reduced blood supply to the affected limb. A worsening of the condition is sometimes seen at the age of 30–50 years, when the desire may be so distressing that the affected individuals might engage in hazardous behaviours like self-amputation.^3,4^

The prevalence of the disorder in the general population is unknown. Although systematic epidemiological and clinical studies on BID are still lacking, BID predominantly affects males and commonly involves the desire to amputate the *left* leg: this suggests a tendency towards left-sided amputations over right-sided ones. However, some affected individuals express a desire for bilateral amputations or the removal of right-sided limbs, highlighting the diverse manifestations of this complex condition.^1-4^

The definition of BID has significantly evolved over recent decades. Initially recognized as apotemnophilia, a paraphilia for amputees and amputation, the interpretation of this condition has broadened considerably. First^5^ introduced the term ‘body integrity identity disorder’, drawing parallels to what was then known as ‘gender identity disorder.’ This new definition was proposed to emphasize the underlying psychological issues, pointing towards a severe disruption in forming a coherent body identity. In 2011, McGeoch and colleagues proposed the term ‘xenomelia’, attributing the desire for amputation to a neurological cause, precisely a distortion in body image linked to dysfunction at the neural level, in the right parietal cortex^6^ (see below).

Although BID is not recognized in the DSM-5, it was officially included in the ICD-11 in 2018.

### Exploring the possible neurological bases of body integrity dysphoria

In recent years, many studies have suggested a possible neurological origin for BID, focusing on registering physiological parameters, such as skin conductance or body temperature or investigating neural anatomical and physiological alterations using brain imaging techniques.

#### Physiological studies

Brang and colleagues^7^ reported an augmented skin conductance response in two individuals with BID when a pinprick was applied below the desired amputation line. Even if the authors did not collect brain structural or functional data, they suggested that BID may arise from congenital dysfunction in the right parietal lobe and its connection with the insula, an area crucial for multisensory integration. They also proposed that the condition may be related to a mismatch between preserved somatosensory processing and a deficient higher-order representation of the body in the superior parietal lobe.^7^

Romano and colleagues^8^ found similar results using noxious stimuli. The authors reported stronger skin conductance responses for stimuli contacting the non-accepted compared with the unaffected body part. They also found a reduced anticipatory response to stimuli approaching the limb affected by the desire for amputation.^8^ These results suggest that BID may inhibit the anticipatory physiological responses to incoming threats. This would turn into an unexpected stimulation when the stimulation touches the limb, which is known to induce stronger pain sensations and physiological reactions.^9^ It is worth noting that this physiological pattern is different from what is observed in complex psychiatric conditions, such as schizophrenia, where electrodermal signals evoked by pain anticipation are preserved.^10^

More recently, Salvato *et al*.^11^ recorded thermal image sequences of circumscribed regions of the limbs’ skin in individuals with BID and found a bilateral decrease in leg temperature when individuals with BID focused their attention on lower limbs. The authors concluded that alteration of the sense of body ownership in BID is associated with abnormal thermoregulatory patterns in response to modulation of attention to body parts.^11^

#### Brain imaging structural studies

To date, only a few studies have investigated the neural basis of this condition using structural or functional neuroimaging techniques.^12-18^ These are summarized in Supplementary Fig. 1A and B.

To provide a more concise and formal summary of this previous literature, we reduced the aforementioned results using a hierarchical clustering meta-analysis made with the software CluB,^19^ with the default parameters described in the original paper. For this purpose, the regional effects illustrated in Supplementary Fig. 1A and B were the raw data for the hierarchical clustering analysis. We applied a very loose data reduction with the only constraint that a cluster should contain at least two peaks of the original raw data and fall in a sphere with a 7 mm diameter. It should be noted that this analysis has illustration purposes only in that, given the paucity of studies, no statistical inference can be made about any regional task effects.

Reductions in grey matter density overlap across multiple brain regions, such as the left inferior frontal gyrus, the right hippocampus and the right primary somatosensory cortex. Additionally, diminished brain activation is evident in the left secondary somatosensory cortex and the right superior parietal lobule, further emphasizing the affected areas (see Supplementary Fig. 1C (left) and Supplementary Table 1A). In contrast, increased grey matter density is observed in the right supramarginal gyrus and the right thalamus (see Supplementary Fig. 1C (right) and Supplementary Table 1B).

Augmented functional activations were reported only in one study and thus not included in the present meta-analysis.

Taken together, these results confirm that BID may be the consequence of the malfunctioning of a distributed network involved in body representation, the so-called body matrix.

There is a *stone guest* among the brain system identified so far, that is, the limbic/affective brain: this seems to play a little, if any, role in BID, a syndrome that, by definition, should be associated with distress in the processing of signals coming from the limb segment felt as exceeding to or not belonging to the body matrix. However, this could be due, in part, to the stimulation procedures adopted so far.

Indeed, the functional tasks described in the previous paragraphs targeted a specific portion of the limb within the to-be-amputated part (e.g. the feet^20^). The monotonous nature of such stimulations and the lack of comparison with a different not to-be-amputated part of the same side have made the protocols above somehow incomplete.

The only two studies considering tactile sites above and below the desired amputation line are characterized by small sample sizes.^16,21^ Moreover, the limb and side for which there was a desire for amputation varied from subject to subject, making the emergence of a systematic difference less likely.

Finally, it should be noted that none of these studies specifically focused their analyses on the moment when the tactile stimulation *crossed* the desired amputation line, as reported by the BID individuals themselves.

#### Aims and predictions

Subjects with BID are adamant not only in indicating which limb they would like to see amputated but also the exact level of where this amputation should occur, generally from well above their knee, at mid of the thigh. This specificity suggests that the chosen boundary holds a significant role in their body representation: neuroimaging offers a crucial window into understanding these alterations by revealing how the brain differentiates between the ‘desired’ and ‘undesired’ parts of the body. By investigating the neural responses to tactile stimuli that cross this self-identified boundary, we can explore whether BID is associated with atypical somatosensory integration, heightened attentional focus on the affected limb or an emotional aversion to certain body parts.

For all these motivations, we devised a dynamic tactile aiming to uncover distinct patterns of brain activity that may reflect either lower-level somatosensory processing or higher-order cognitive and emotional mechanisms.

We had a few predictions in mind. On the one hand, we anticipated that dynamic tactile stimulation in individuals with BID could reveal abnormal brain activations within the somatosensory and related integration areas, reflecting the underlying altered processing of somatosensory signals, as previously observed.^20,21^ This should be specific for the to-be-amputated limb segment, emphasizing when the border of the two segments is crossed, and for a specific direction of stimulation (e.g. moving from the ‘healthy’ to the ‘to-be-amputated’ part of the limb).

On the other hand, given the peculiarity of our tactile stimulation, we foresaw leg and group-specific activations at the level of brain regions also involved with affective/emotional regulation. Indeed, as we will further explain in the next section, the stimulation was applied around the point of the desired amputation, involving a slow movement towards or far from this specific location. This region holds strong personal and emotional relevance for individuals with BID. Consequently, tactile input here may engage affective and emotional processing networks due to its heightened personal significance.

Moreover, the task required participants to actively detect when the stimulus crossed the line of their desired amputation, rather than passively experiencing touch as in previous studies.^20,21^ This focused attention and anticipation can engage brain regions involved in emotional and affective processing. Based on these considerations, we anticipate potential alterations in brain activity within regions associated with affective and emotional regulation.

If such prediction could be confirmed, BID could finally be associated not only with a dysfunction of a body matrix but also with pathological affective dysregulation. As the reader shall see, these predictions were only in part met, making perhaps our observations more revealing.

## Material and methods

### Participants

Individuals with BID were recruited via online advertisements and through participant referrals (word-of-mouth). All participants provided written informed consent, and the study was approved by the Local Ethics Committee of the ASL of Milan (Comitato Etico Azienda Sanitaria Locale Città di Milano). The research was conducted in accordance with the ethical principles outlined in the 1964 Declaration of Helsinki.

The final sample included nine male participants with a long-standing desire for the amputation of their left leg above the knee (mean age = 42.11 ± 9.16 years; age range = 34–64; mean years of education = 15.33 ± 2.55; range = 13–18), along with 14 healthy control participants (mean age = 38 ± 9.20 years; age range = 26–53; mean education = 15.50 ± 3.98 years; range = 8–18; see Table 1). This sample was also partially described in the study of Gandola *et al*.^20^ and Saetta *et al*.^16,17^ The sample size was based on these previous studies.

**Table 1:** Demographic and clinical features of participants with BID

Participants with BID	Demographic features		BID features		Mean scores on the Zurich Xenomelia Scale (SD)^a^			
	Age	Education	Limb	Side	Pure amputation desire	Erotic attraction	Pretending behaviour	Total scale scores
P1	42	13	Leg	Left	4.50	4.25	5.00	4.58
P2	36	13	Leg	Left	5.25	3.25	3.00	3.83
P3	48	18	Leg	Left	6.00	3.50	4.00	4.50
P4	34	18	Leg	Left	5.25	2.75	3.25	3.75
P5	37	18	Leg	Left	6.00	6.00	4.75	5.58
P6	41	13	Leg	Left	5.75	4.50	5.00	5.08
P7	39	18	Leg	Left	6.00	4.25	5.50	5.25
P8	64	13	Leg	Left	6.00	5.50	4.50	5.33
P9	38	14	Leg	Left	6.00	3.75	3.00	4.25

^a^Ranges 1–6.

### Clinical assessment

All participants with BID reported experiencing considerable psychological distress and dedicating several hours daily to thoughts about amputation, as well as searching for BID- and amputation-related information online. They consistently described the condition as having been present since early childhood, often stating that ‘BID was always there’. Despite their strong conviction that their condition stemmed from a neurological basis, none had sought surgical intervention or attempted self-amputation. Clinical interviews confirmed that all individuals met the diagnostic criteria for BID as outlined in the ICD-11 (code 6C21; https://icd.who.int/browse/2025-01/mms/en#256572629).

Neurological assessments were conducted using standard clinical procedures to evaluate motor function as well as somatosensory and visual perception in response to both unilateral and bilateral stimuli.^22^ Psychiatric evaluations were carried out through clinical interviews under the supervision of P.B. The desire for limb amputation was systematically assessed using the Zurich Xenomelia Scale^23^ (Table 1).

### Functional MRI experiment

The functional MRI (fMRI) experiment involved a manual dynamic tactile stimulation of the ‘affected’ and the ‘unaffected’ limbs above and below the line of the desired amputation and its corresponding level contralaterally.

For BID individuals, we first identified and marked the line of desired amputation on the affected limb; the same line on the contralateral limb was also identified and marked with paper tape. We calculated the mean position of the BID individuals’ point of desired amputation (23.4 ± 2.1 cm, starting from the femoral head). This position was then used to study the healthy controls with their thighs marked with paper tape at that same level.

Participants were then instructed to focus on the perceived tactile sensations. An experimenter (M.G. or L.Z.) continuously touched the affected and unaffected limb with her index finger at a speed of about 2 cm/s.

The stimulation was applied starting from (i) 10 cm above the indicated point and moving down to 10 cm below this point or (ii) 10 cm below the point and moving up to 10 cm above this point (see Supplementary Fig. 2).

Participants indicated when they perceived the experimenter's index finger passing over the desired amputation line by pressing a button with the index finger of the ipsilateral hand to the stimulation using response boxes available in the MRI equipment. During the stimulation of the healthy limb, BID individuals had to press the button when passing over the level marked with an elastic band. The same request was made to the healthy controls (for both limbs).

We repeated the stimulation 32 times for each leg (16 for each direction of stimulation).

### Functional MRI data acquisition

fMRI data were acquired using a 1.5 Tesla GE Signa HD-XT scanner, utilizing an echo planar imaging (EPI) gradient-echo sequence with the following parameters: flip angle of 90°, echo time (TE) of 60 ms, repetition time (TR) of 3000 ms, field of view (FOV) of 240 × 240 mm and a matrix resolution of 64 × 64. Each volume included 35 interleaved axial slices, aligned along the AC–PC plane, with a slice thickness of 5 mm and no interslice gap. Volumes were captured at 3-s intervals, and a total of 450 volumes were obtained per participant. The initial 10 volumes, corresponding to task instruction periods, were excluded from further analysis.

In addition, a high-resolution anatomical scan of the entire brain was collected for each subject using a 3D spoiled gradient-recalled sequence. This scan was acquired with a flip angle of 20°, TE of 2.92 ms, TR of 9.2 ms and a matrix size of 256 × 256. The slices had a thickness of 1 mm, no interslice gap and an isotropic voxel size of 1 × 1 × 1 mm. The anatomical scan comprised 154 continuous slices acquired on oblique sections parallel to the AC–PC line to cover the entire brain volume.

### Preprocessing

Following image reconstruction, raw data were visualized and converted from DICOM to NIFTI format using the dcm2nii tool within the MRIcron software package. All subsequent analyses were conducted in MATLAB version 8.1 (MathWorks, Natick, MA, USA) utilizing the Statistical Parametric Mapping software (SPM12, Wellcome Department of Imaging Neuroscience, London, UK). Initial inspection of the images focused on identifying motion artefacts and any obvious anatomical abnormalities, after which they were manually aligned to the anterior commissure.

fMRI scans were then realigned to the first scan to correct for movement during acquisition. The realigned images were stereotactically normalized to the Montreal Neurological Institute template space to enable group-level analyses. During this process, voxel dimensions were resampled to 2 × 2 × 2 mm.

To enhance the signal-to-noise ratio, the normalized and realigned images were smoothed using a Gaussian kernel with a full width at half maximum of 10 × 10 × 10 mm.^24,25^

### Statistical analysis of the functional MRI data

A convolution with a canonical haemodynamic response function then analysed the blood-oxygen-level-dependent (BOLD) signal associated with each experimental condition.^26^ The global differences in fMRI signals were removed using proportional scaling for all the voxels on the global counts. High-pass filtering (128 s) was used to remove artefactual contributions to the fMRI signal, such as physiological noise from cardiac and respiration cycles.

The BOLD signal corresponding to each event was modelled through convolution with a canonical haemodynamic response function.^26^

*First-level fixed-effect analysis.* An initial fixed-effects block analysis was conducted at the individual subject level to model the BOLD response associated with each event, relative to an implicit baseline. To account for motion-related artefacts, the realignment parameters were included as regressors in the design matrix. This analysis specifically examined the BOLD responses elicited by tactile stimulation of different regions of each leg, namely, areas located above, below or intersecting the intended amputation line, as well as the direction in which the stimulation was applied.

*Second-level random-effect analysis*. The resulting contrast images were incorporated into separate second-level full factorial ANOVAs, in line with random-effects analysis procedures, to enable generalization of the statistical findings to the broader population.^27^ The data were analysed within a factorial design framework to address the following research questions:

***Question 1.*** Topography of the stimulation (general). Do individuals with BID show altered neural activations in response to tactile stimulation, especially when considering the specific leg and the specific segment of the leg? *Factors considered*: Group (controls versus BID), side (right versus left leg) and part of the leg (above or below the desired amputation line).

***Question 2.*** Direction of the stimulation. Do individuals with BID show altered neural activations in response to tactile stimulation, especially when considering the specific leg and the direction of stimulation with respect to the desired line of amputation? *Factors considered*: Group (controls versus BID), side (right versus left leg) and direction of stimulation (moving towards versus away from the desired amputation line).

***Question 3.*** Amputation line. Do individuals with BID show altered neural activations when the tactile stimulation crosses the desired amputation line compared with control subjects, especially when considering the direction of stimulation? *Factors considered*: Group (controls versus BID), side (right versus left leg) and direction of stimulation (moving towards versus away from the body part to be amputated).

All reported results survive a whole-brain cluster-level family-wise error rate (FWER) correction for multiple comparisons (pFWER < 0.05). The voxel-wise threshold applied to the statistical maps before the cluster-wise correction is *P* < 0.001 uncorrected, as recommended elsewhere.^28^ The peaks that survive the statistical threshold *P* < 0.05 whole-brain FWER-corrected voxel-wise (peak level) are also reported in the tables. Accordingly, all results described were corrected for multiple comparisons using state-of-the-art approaches.

To assess the extent to which the scores at the three subscales of the *Zurich Xenomelia Scale*, we run parametric or non-parametric correlation analyses (depending on data distribution) between these scores and the brain response at the peak of maximum difference with healthy controls.

### Neurosynth decoding of the brain functional abnormalities

Besides typical forward inferences based on the experimental design and interaction of factors, we also considered the strength of associations between the actual statistical maps of our analyses and neuroscientific semantics as indexed by specific keywords. Specifically, the BID-related functional abnormalities clusters were loaded into the Neurosynth.org database and analysed using the ‘decoder’ function (https://neurosynth.org/decode/).^29^ The decoder function of Neurosynth allows one to retrieve the Pearson correlation of the keywords most associated with the input image, containing the clusters identified by our fMRI analyses based on the NeuroVault repository. The *r*-value related to each keyword reflects the correlation across all voxels between the input map and the map associated with a particular keyword in NeuroVault. In other words, while not replacing the typical forward inferences in the current study, the quantitative associations that Neurosynth returned may provide valuable information for our discussion.

## Results

***Question 1.*** Both individuals with BID and healthy controls showed significant activations of the contralateral primary and secondary somatosensory cortices in response to tactile stimulation of each leg (see Supplementary Fig. 3 and Supplementary Tables 2A and B).

**Figure 1:**
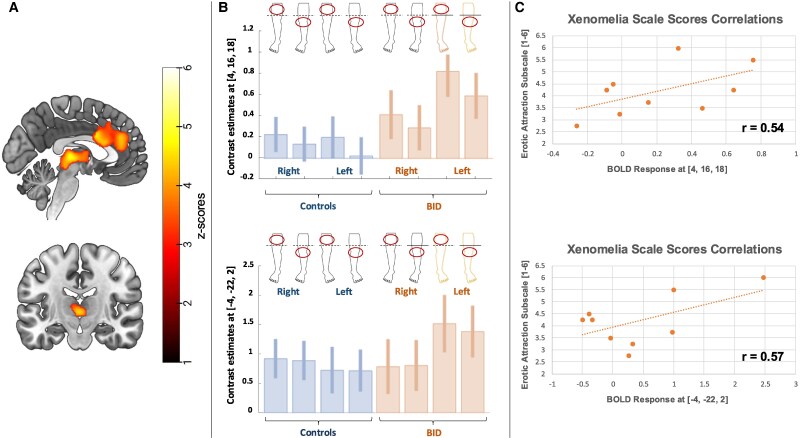
**fMRI results: topography of the stimulation.** (**A**) Full factorial ANOVA (*N* = 23). Brain regions showing a significant group-by-side interaction, with increased activations recorded in individuals with BID, particularly in response to the stimulation of the left leg (independently from the part of the leg). (**B**) Full factorial ANOVA (*N* = 23). Plot of the haemodynamic response recorded in the local maxima of the displayed clusters: anterior cingulum, [4, 16, 18], *Z*-score: 4.69, (*P* < 0.001 at peak level, *P* < 05 family-wise error corrected at cluster level); thalamus [−4, −22, 2], *Z*-score: 4.60, (*P* < 0.001 at peak level, *P* < 0.05 family-wise error corrected at cluster level). (**C**) Scatterplot of the correlation analysis (*N* = 9) between the haemodynamic response and BID individuals’ score at the Zurich Xenomelia Scale (erotic attraction subscale; min = 1; max = 6): anterior cingulum, [4, 16, 18], Pearson’s coefficients: 0.54; thalamus [−4, −22, 2], Pearson’s coefficients: 0.57. Each data point represents a BID individual’s BOLD response plotted against the corresponding score on the subscale.

However, we also observed a significant group-by-side interaction in an extensive cortical and subcortical network, including the orbitofrontal cortex, the anterior cingulum, the insula, the thalamus, the caudate, the putamen, the nucleus accumbens and the midbrain. These regions were more active in response to the tactile stimulation of the left leg in individuals with BID, independently from the part of the leg (i.e. above and below the amputation line, see Fig. 1 and Table 2). Importantly, these group-by-side interaction effects were well outside the canonical sensorimotor network observed in previous studies (see Supplementary Fig. 4).

**Table 2:** Group * leg interaction: left leg > right leg BID > controls (tactile stimulation above and below the amputation line)

Brain region (BA)	Left hemisphere				Right hemisphere			
	*x*	*y*	*z*	*Z*-score	*x*	*y*	*z*	*Z*-score
Anterior orbital gyrus (11)	−18	40	−10	3.27	20	46	−14	3.23
Superior frontal gyrus (11)	-	-	-	-	22	52	−2	3.78
Superior frontal gyrus medial (11) and medial orbital (10)	−8	24	42	3.64	18	50	4	3.36
	-	-	-	-	10	44	−4	3.10
Anterior cingulate cortex (24/32)	−2	38	24	4.14	4	16	18	4.69^a^
	-	-	-	-	0	8	30	4.53^a^
	-	-	-	-	0	38	18	4.18
	-	-	-	-	14	42	8	3.70
	-	-	-	-	10	42	10	3.66
Posterior orbital gyrus (11)	−26	26	−20	4.19	-	-	-	-
	−30	30	−20	4.09	-	-	-	-
Insula	−28	22	6	3.71	-	-	-	-
Thalamus	−4	−22	2	4.60^a^	6	−2	8	3.75
	-	-	-	-	2	−4	8	3.74
Caudate	-	-	-	-	18	22	−10	4.46^a^
Putamen	−26	12	0	4.49^a^	30	14	4	3.70
	−22	−4	8	4.45^a^	-	-	-	-
	−24	14	−4	4.36	-	-	-	-
	−16	16	−8	3.85	-	-	-	-
Nucleus accumbens	-	-	-	-	6	8	−8	3.47
	-	-	-	-	4	4	−8	3.44
Midbrain	−4	−28	−8	3.97	-	-	-	-

^a^FWER 0.05 correction (voxel level).

The decoding of these brain functional abnormalities based on Neurosynth showed that the functional domains that best correlated with this anatomical pattern were ‘reward’ (Correlation coefficient: 0.19), ‘gain’ (Correlation coefficient: 0.18), ‘incentive delay’ (Correlation coefficient: 0.18), ‘anticipation’ (Correlation coefficient: 0.15), ‘losses’ (Correlation coefficient: 0.152) and ‘pain’ (Correlation coefficient: 0.12).

These patterns showed a moderate positive correlation with the Zurich Xenomelia Scale (erotic attraction subscale): Pearson’s coefficients: 0.54 for the anterior cingulum [4, 16, 18] and 0.57 for the thalamus [−4, −22, 2], see Supplementary Table 3.

**Figure 2:**
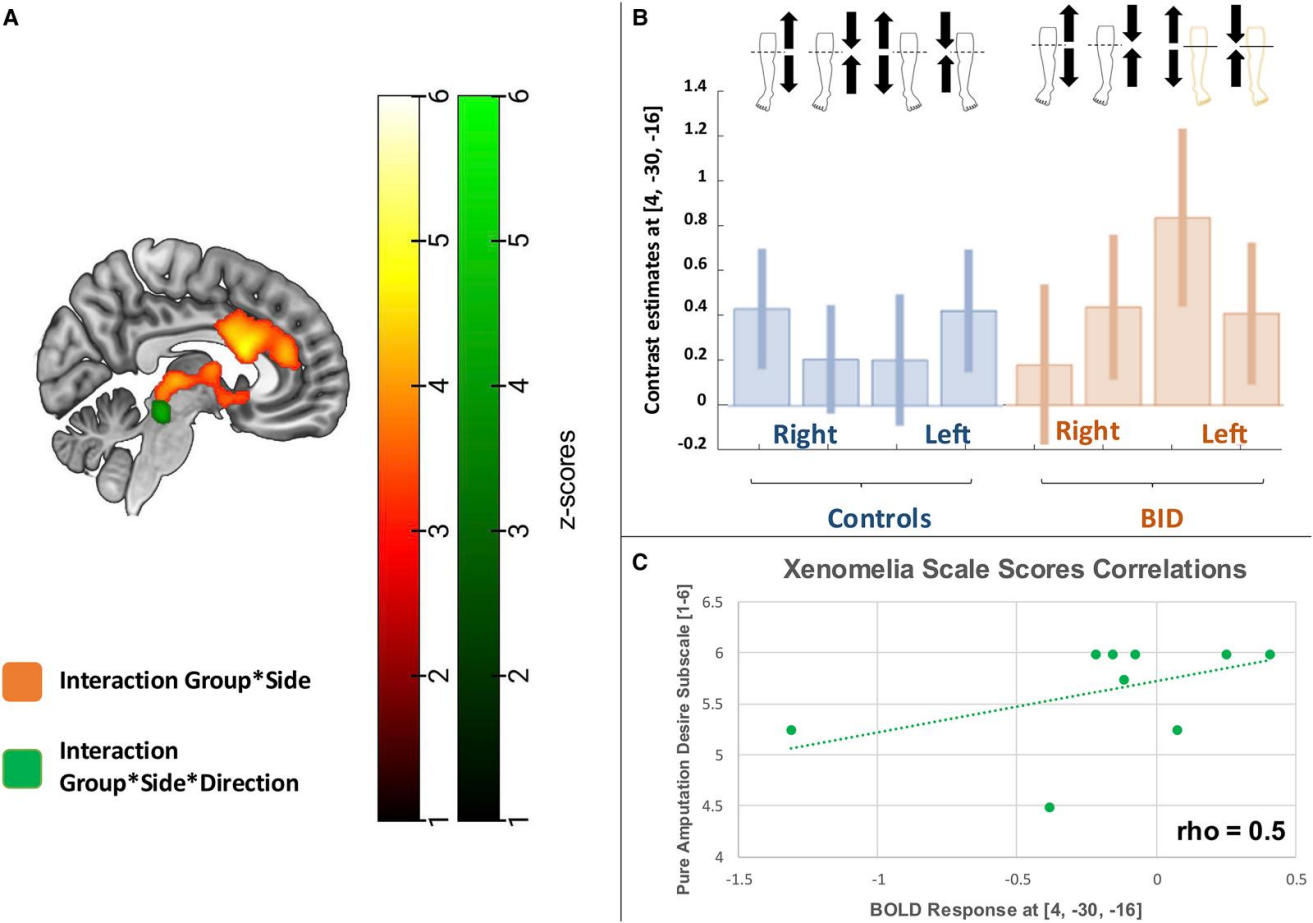
**fMRI results: direction of the stimulation.** (**A**) Full factorial ANOVA (*N* = 23). Brain regions showing a significant group-by-side interaction, with increased activations recorded in individuals with BID, particularly in response to the stimulation of the left leg (independently from the part of the leg). (**B**) Full factorial ANOVA (*N* = 23). Plot of the haemodynamic response recorded in the local maxima of the displayed cluster: periaqueductal grey [4, −30, −16]; *Z*-score: 4.30 (*P* < 0.001 at peak level, *P* < 0.05 family-wise error corrected at cluster level). (**C**) Scatterplot of the correlation analysis (*N* = 9) between the BID individuals’ haemodynamic response and their score at the Zurich Xenomelia Scale (pure amputation desire subscale; min = 1; max = 6): periaqueductal grey, [4, −30, −16], Spearman’s coefficient: 0.5. Each data point represents a BID individual’s BOLD response plotted against the corresponding score on the subscale.

***Question 2.*** We observed a significant group-by-side by direction interaction at the level of the midbrain near the periaqueductal grey, with increased activations recorded in individuals with BID, particularly in response to the stimulation of the desired amputation line of the left leg after the stimulation has passed the line of the desired amputation (see Fig. 2). The functional domain terms that best correlated with this anatomical pattern, according with Neurosynth, were ‘pain’ (Correlation coefficient: 0.1), ‘sustained’ (Correlation coefficient: 0.16), ‘ratings’ (Correlation coefficient: 0.13) and ‘chronic pain’ (Correlation coefficient: 0.08).

These patterns showed a moderate positive correlation with the Zurich Xenomelia Scale (amputation desire subscale) in the periaqueductal grey (Spearman’s coefficient = 0.5 for the peak [4, −30, −16], see Supplementary Table 3).

**Figure 3:**
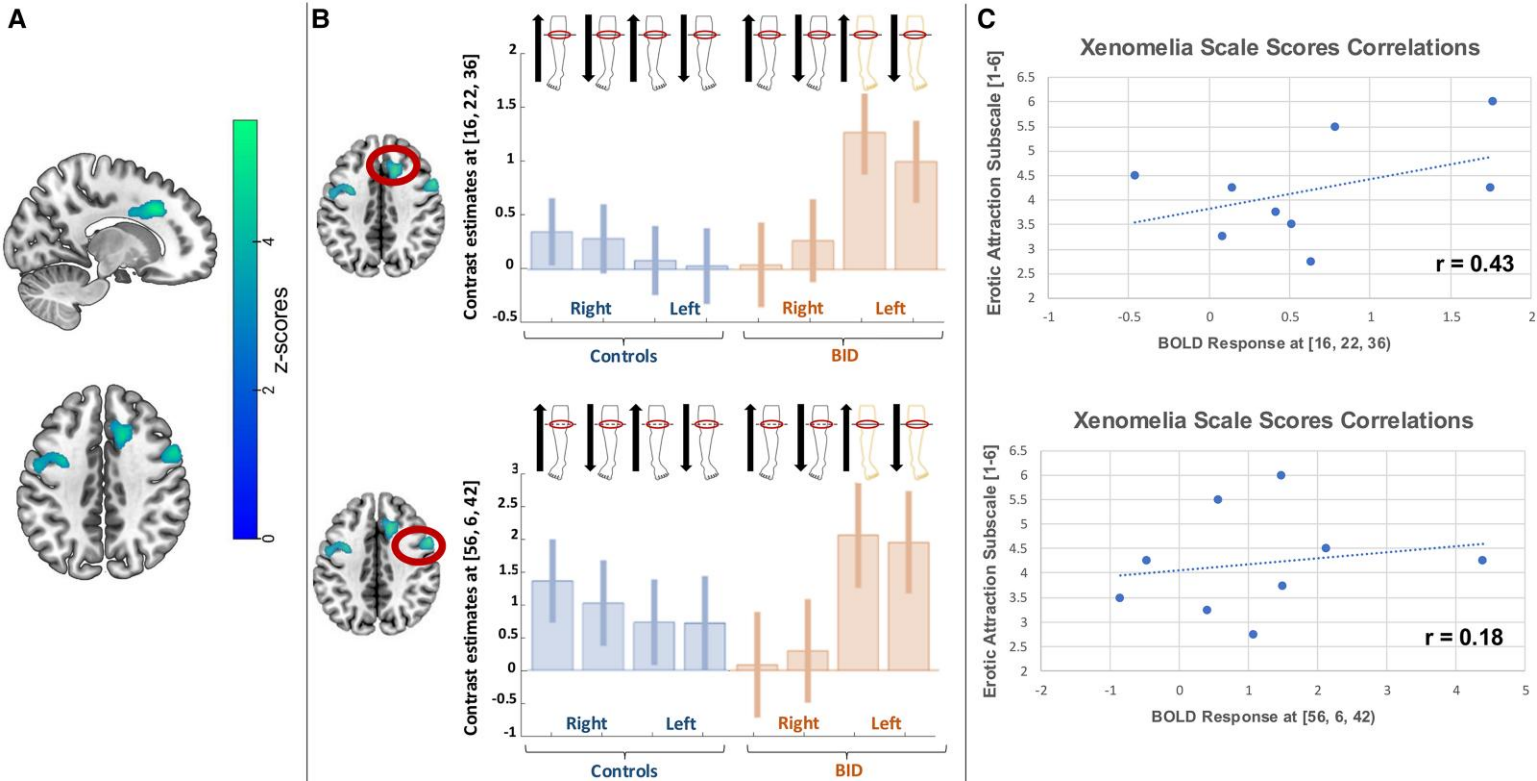
**fMRI results: amputation line.** (**A**) Full factorial ANOVA (*N* = 23). Brain regions showing a significant group-by-side interaction, with increased activations recorded in individuals with BID, particularly in response to the stimulation of the desired point of amputation (independently from the direction). (**B**) Full factorial ANOVA (*N* = 23). Plot of the haemodynamic response recorded in the local maxima of the displayed clusters: middle cingulum [16, 22, 33], *Z*-score: 5.21, (*P* < 0.001 at peak level, *P* < 05 family-wise error corrected at cluster level); precentral gyrus [56, 6, 42], *Z*-score: 4.75, (*P* < 0.001 at peak level, *P* < 0.05 family-wise error corrected at cluster level). (**C**) Scatterplot of the correlation analysis (*N* = 9) between the BID individuals’ haemodynamic response and their score at the Zurich Xenomelia Scale (erotic attraction subscale; min = 1; max = 6): middle cingulum [16, 22, 33], Pearson’s coefficient = 0.43; precentral gyrus [56, 6, 42], Pearson’s coefficient = 0.17. Each data point represents a BID individual’s BOLD response plotted against the corresponding score on the subscale.

***Question 3.*** We observed a significant group-by-side interaction in the precentral gyri, the anterior cingulum and the superior and middle temporal gyrus. These regions were more active in response to the tactile stimulation *over* the left leg desired amputation line, independently from the direction of stimulation (see Fig. 3 and Table 3).

**Table 3:** Group * leg interaction: left leg > right leg BID > controls (tactile stimulation over the amputation line)

Brain region (BA)	Left hemisphere				Right hemisphere			
	*x*	*y*	*z*	*Z*-score	*x*	*y*	*z*	*Z*-score
Middle cingulate (32)/Superior frontal gyrus medial (9)	-	-	-	-	16	22	36	5.21^a^
	-	-	-	-	16	26	34	5.11^a^
	-	-	-	-	16	4	32	3.61
	-	-	-	-	8	40	34	3.29
	-	-	-	-	4	42	30	3.21
Precentral gyrus (6)	−34	2	42	4.40^a^	56	6	42	4.75^a^
	−52	−2	36	3.76	-	-	-	-
	−48	−6	50	3.30	-	-	-	-
	−40	4	32	3.24	-	-	-	-
Superior and middle temporal gyrus (21–22)	−64	−22	−12	4.91^a^	-	-	-	-
	−64	−36	6	3.54	-	-	-	-
	−56	−24	0	3.30	-	-	-	-

^a^FWER 0.05 correction (voxel level).

The Neurosynth functional domain terms that best correlated with this anatomical pattern were ‘conflict’ (Correlation coefficient: 0.16) and ‘Stroop’ (Correlation coefficient: 0.09).

We observed a moderate correlation between the erotic attraction subscale of the Zurich Xenomelia Scale in the middle cingulum: Pearson’s coefficient = 0.43 for the peak [16, 22, 33], see Supplementary Table 3. Only a very weak correlation was present for the premotor cortex: Pearson’s coefficient = 0.18 for the peak [56, 6, 42].

## Discussion

We recognize our body as belonging to us as much as we monitor its position and its movement in space.^30^ Both the sense of body ownership and self-location, in fact, make our sense of self-awareness coherent. Different disorders can disrupt these processes, leading to significant distortions in body representation and sense of body ownership: somatoparaphrenia (i.e. misattributing one's body parts to others^31-33^), misoplegia (i.e. intense dislike or hatred for one's own body parts^34^), asomatognosia (i.e. lack of awareness of a body part, making it feel as though it has disappeared from consciousness^35^) or pathological embodiment (i.e. the inclusion of others’ limbs into our body schema^36^). All these disorders make it clear—more than any illusion in normal subjects—that our body representation and sense of body ownership are complex and vulnerable at the same time.

Although BID and these conditions may be considered sisters and brothers due to the general impairment of the body representation that makes them similar, there are relevant differences: first and foremost, even if somatoparaphrenia may radicalize into misoplegia for the left limb, a patient with this disorder would never even dream to amputate his/her limb although paralysed. Secondly, BID is characterized by the lack of macroscopic brain damage, which has motivated functional imaging studies, including this one. Finally, the limb for which amputation is typically intact in BID. Nonetheless, it causes a distressing sense of mismatch between the current and the desired body: typically, individuals with BID claim that that limb, although recognized as belonging to their body, is nevertheless felt as an extra part of it.

Coming to the similarities, ‘left-sided’ BID is more frequent,^2^ mirroring neuropsychological symptoms associated with right brain damage, mainly in the right parietal lobe, insula and basal ganglia.^32,37^ This suggests a neurological aetiology also for BID.

Recently, we have shown that BID is associated with abnormal neural functional connectivity in a neural network, including two main regions of the motor and somatosensory system: the right paracentral lobule and the right superior parietal lobule.^16^ Task-based fMRI data on a subsample of the same individuals indicated that these regions are also less active in BID in response to a monotonic tactile stimulation of the left foot.^20^

In the present study, we rather used a dynamic tactile stimulation of the lower limbs with a task that emphasized the attentional stance to the very point where the BID individuals wanted their leg to be amputated and, for a control, the same position on the right thigh.

Our results showed the expected contralateral brain activations in the sensorimotor brain regions in both healthy controls and subjects with BID. However, we also observed altered neural activations in individuals with BID not only in areas concerned with sensory processing but also in regions associated with reward encoding and pain (see also^18^). The identification of this diverse functional pattern suggests that BID is a complex condition not only limited to a disorder of body representation; the same findings rather offer a neurodysfunctional basis for what is mainly observed at a clinical level as the complaint of a sense of extraneity for one limb.

We discuss our results by addressing the three main questions introduced at the beginning of the paper that guided our analyses and that we described in the results section. All comments refer to brain patterns associated with stimulations to the left leg.

### Question 1. Topography of the stimulation (general). Do individuals with BID show altered neural activations in response to tactile stimulation, especially when considering the specific leg and the specific segment of the leg?

Our results show a significant pattern of hyperactivations in individuals with BID in response to the dynamic tactile stimulation of the left leg affected by the desire for amputation.

This aberrant neural activity involved the orbitofrontal cortex, the anterior cingulum, the thalamus, the basal ganglia and the midbrain. These regions are well outside the typical network involved in somatosensory processing observed in our previous study^20^ (Supplementary Fig. 4). The dynamic nature of the touch we have used here makes this stimulation much more like, even though not canonically so, the so-called *affective touch*,^38^ representing the so far missing other side of the coin in the characterization of somatosensory processing in BID.

It is important to clarify that our stimulation was not specifically designed to optimize C-Tactile fibre activation, nor can we assess whether it was perceived as pleasant by individuals with BID or if their perception differed from that of the control group, given the absence of pleasantness ratings. Nevertheless, our stimulation shares key characteristics with affective touch, particularly its dynamic nature and slow velocity. In our view, these features—combined with the specific demands of our task, the spatial localization and the dynamics of the stimulation—likely contributed to the emergence of altered brain dynamics involving regions associated with affective and emotional processing.

We suggest that increased limbic activity may indicate a heightened affective response to the sensory dynamic and continuous input, especially concerning the discomfort or incongruity felt for the leg of the desired amputation. The brain might also engage in compensatory mechanisms to deal with the perceived ‘error’ in body integrity. This could manifest as heightened activity in the limbic system as it processes complex emotions related to the perceived ‘extra’ parts of the body.

This result was similar when the stimulation of the left leg was both above and below the desired line of amputation. This suggests that the proximal part of the limb is also felt not to be right as long as it is connected with the segment perceived in excess. A study on BID amputees could clarify this issue.

### Question 2. Direction of the stimulation. Do individuals with BID show altered neural activations in response to tactile stimulation, especially when considering the specific leg and the direction of stimulation with respect to the desired line of amputation?

When the stimulation had just passed the line of the desired amputation, there were increased activations in the periaqueductal grey, specifically for the left thigh. Attentional mechanisms have a modulatory effect on subjective pain perception, attention being a critical component of the pain experience.^39^

The periaqueductal grey, along with the anterior cingulate cortex, is part of a primary pain modulatory pathways that regulate facilitatory or inhibitory modulation of nociception through the spinal cord dorsal horn.^40^ Thus, the effect described here could be due to attentional phenomena: these were most likely at their peak the very moment the tactile stimulation had passed the line of desired amputation, a moment that we may have had a maximal implicit psychic pain effect—even if not explicitly reported by the experimental subjects.

### Question 3. Amputation line. Do individuals with BID show altered neural activations when the tactile stimulation crosses the desired amputation line compared with control subjects, especially when considering the direction of stimulation?

Our data also indicate that the line of desired amputation has a special status when monitoring somatosensory stimuli: the motor response generated when the tactile stimulation crossed the line of desired amputation was associated with increased activity in the frontal lobe, in the anterior cingulum and in premotor regions. A strong activation in the premotor cortex is a hallmark finding in all ready-steady-go paradigms in non-human primates^41^ as much as in humans.^42^ Hence, augmented recruitment of premotor regions may be related to a stronger ‘motor alert’ boosted by the extra-motivation felt by the participants with BID.

On the other hand, as widely known, the anterior cingulate cortex is involved in emotional and conflict regulation.^43^ When subjects with BID perceived that the amputation line had been crossed, they might have experienced a unique, intense emotional state to suggest that this border has a special status in the BID individuals’ minds.

### How *painful* or rewarding is (to live with a condition like) BID?

This study, for the first time, brings to the centre stage the limbic brain and the reward system in BID rather than a set of brain regions primarily associated with a cartographic mapping of the body. Admittedly, this finding is not fully aligned with our predictions, which were more towards the emotional brain, surely intertwined with the reward system but not fully corresponding with it. For example, a stronger activation of the amygdala in BID would have been, if confirmed, a much-anticipated benchmark for our initial prediction. However, most of the patterns of brain activity that did tease apart subjects with BID from the healthy controls pointed to the dimension of *reward* and *pain*, as indicated by quantitative reverse inference offered by Neurosynth.

Would the collection of subjective affective experiences during tactile stimulation have strengthened the interpretation of our fMRI data? This is somewhat questionable as the introduction of a meta-cognitive introspective component in the task would have dramatically changed the nature of our study, which was aimed at capturing implicit neural responses rather than those associated with meta-cognitive introspection. This decision had both advantages and disadvantages. The primary advantage was the ability to identify a more readily distinguishable neural pattern, as implicit responses—being less susceptible to bias than explicit ones—are often more reliable across various experimental psychology paradigms (e.g. skin conductance changes in response to noxious stimuli approaching the left leg in BID, compared with explicit reports showing no difference between left and right leg—see^8^; for other sources of evidence about the distinction between explicit and implicit measures see, for example^44,45^). In the absence of direct subjective reports during fMRI scanning, we relied on the probability-based decoding function of the Neurosynth database, which offers an objective, data-driven approach to decoding rather than anecdotal interpretations. Our findings also revealed a positive correlation between neural activations in mesolimbic regions and the erotic attraction subscale of the Zurich Xenomelia Scale, reinforcing the relevance of affective dimensions in our findings. The downside of not having explicit reports of the participants’ mental states is the difficulty of making a direct link between the neural responses and the participants’ lived experiences. Collecting these reports, at least at the end of the fMRI scans, could have informed the interpretation of the results and should be considered in future studies.

Our findings, besides offering a new view on a hidden side of BID, ‘makes sense’ as much as individuals with BID might experience a vivid reaction when they are stimulated around the line where they would like to be amputated. A dysfunctional connection of sensory pathways (visual, taste) with the reward system is a hallmark of most psychopathological conditions characterized by obsessions and dependence.^46^

Somatic pain and acute emotional distress were not explicit features in the behaviour and reports of our BID individuals during or following our fMRI study. Yet, BID individuals are known for dull psychic suffering for their body configuration, something that does not translate into the experience of an explicit somatic pain and yet it could always be there, particularly when attention is drawn to the body segment lamented as an exceeding one.

As much as correlation is not causation, the demonstration of profound neurophysiological effects in specific regions of the reward/pain systems of BID individuals suggests that this pathological condition is not a mere cognitive construct, deserving clinical attention rather than sceptical disbelief. Thus, the preliminary signs of a correlation found primarily with the erotic attraction subscale and the brain responses during the dynamic touch experience may make the association between the observed hyperactivations and the reward concept even more plausible.

It is worth recalling that a mere generic sexual arousal during our stimulation cannot account for this finding, as the correlation was found for the differential activation for the left and the right limbs in individuals with BID. It remains to be seen whether the aspect of reward that correlates with brain function is truly erotic in nature or is rather connected with a latent collinear variable in the domain of reward, like, for example, the concept of arousal.

To conclude, our evidence suggests that BID is not due to a pure impairment of the body representation explained as a hill-organized cartography of the body in the brain; instead, it also seems deeply intertwined with the salience of anything happening to the to-be-amputated limb. The connection with altered processing with the reward system may offer a unifying factor for the BID phenomenology that, in some cases, affects body segments differently from the more frequently involved left lower limb considered in this study. Hence, these results seem to support the notion that BID might be driven by complex neural interactions that blend sensory (mis)perceptions with strong affective components, possibly overlearned and pathologically reinforced through experience.

Many aspects of this condition are still ambiguous and deserve further studies: for example, longitudinal studies in subjects who had their left leg amputated may help clarify whether the amputation can reconcile the somatosensory brain with the limbic one. If so, this would add some ground truth to our interpretations.

It is important to highlight that, while your study provides evidence of altered neural responses in BID, distinguishing whether these responses are causal, consequential or compensatory remains a challenge that cannot be settled within our study. Indeed, dysfunctions in limbic/reward-related circuits may predispose individuals to BID, possibly leading to an abnormal integration of body ownership and affective processing. This would align with theories suggesting that BID has a neurodevelopmental origin, with early disruptions in body representation leading to later affective reinforcement.

On the other hand, long-term distress and preoccupation with amputation might have shaped these brain responses over time. The persistent attention to the ‘excessive’ limb and associated affective reinforcement could have led to experience-dependent plasticity, modifying reward and sensorimotor networks. This is a plausible scenario, given that obsessive preoccupation towards the ‘unwanted’ limb is a hallmark of BID. Finally, increased mesolimbic and premotor activations could be an attempt to regulate distress or to enhance sensory monitoring of the affected limb. This could explain why the anterior cingulate and premotor cortices are particularly active regions involved in both conflict monitoring and preparatory motor actions. Altogether, this study adds to the understanding of BID as a multifaceted condition rooted in both perceptual and affective neural mechanisms. Future research should aim to clarify the developmental trajectory and plasticity of these networks.

## Supplementary Material

fcaf209_Supplementary_Data

## Data Availability

The data underlying this article will be shared on reasonable request to the corresponding author. No specific codes were generated and used in this work.

## References

[1] Fornaro S, Patrikelis P, Lucci G. When having a limb means feeling overcomplete. Xenomelia, the chronic sense of disownership and the right parietal lobe hypothesis. Laterality. 2021;26(5):564–583. 33373552 10.1080/1357650X.2020.1866000

[2] Brugger P, Lenggenhager B, Giummarra MJ. Xenomelia: A social neuroscience view of altered bodily self-consciousness. Front Psychol. 2013;4:204. 23630513 10.3389/fpsyg.2013.00204 PMC3634160

[3] Sedda A . Body integrity identity disorder: From a psychological to a neurological syndrome. Neuropsychol Rev. 2011;21(4):334–336. 22071988 10.1007/s11065-011-9186-6

[4] Sedda A, Bottini G. Apotemnophilia, body integrity identity disorder or xenomelia? Psychiatric and neurologic etiologies face each other. Neuropsychiatr Dis Treat. 2014;10:1255–1265. 25045269 10.2147/NDT.S53385 PMC4094630

[5] First MB . Desire for amputation of a limb: Paraphilia, psychosis, or a new type of identity disorder. Psychol Med. 2005;35(6):919–928. 15997612 10.1017/s0033291704003320

[6] McGeoch PD, Brang D, Song T, Lee RR, Huang M, Ramachandran VS. Xenomelia: A new right parietal lobe syndrome. J Neurol Neurosurg Psychiatry. 2011;82(12):1314–1319. 21693632 10.1136/jnnp-2011-300224

[7] Brang D, McGeoch PD, Ramachandran VS. Apotemnophilia: A neurological disorder. NeuroReport. 2008;19(13):1305–1306. 18695512 10.1097/WNR.0b013e32830abc4d

[8] Romano D, Sedda A, Brugger P, Bottini G. Body ownership: When feeling and knowing diverge. Conscious Cogn. 2015;34:140–148. 25955181 10.1016/j.concog.2015.04.008

[9] Brown CA, Seymour B, Boyle Y, El-Deredy W, Jones AKP. Modulation of pain ratings by expectation and uncertainty: Behavioral characteristics and anticipatory neural correlates. Pain. 2008;135(3):240–250. 17614199 10.1016/j.pain.2007.05.022

[10] Linnman C, Coombs G, Goff DC, Holt DJ. Lack of insula reactivity to aversive stimuli in schizophrenia. Schizophr Res. 2013;143(1):150–157. 23201307 10.1016/j.schres.2012.10.038 PMC3540134

[11] Salvato G, Zapparoli L, Gandola M, et al Attention to body parts prompts thermoregulatory reactions in body integrity dysphoria. Cortex. 2022;147:1–8. 34991060 10.1016/j.cortex.2021.11.016

[12] Blom RM, van Wingen GA, van der Wal SJ, et al The desire for amputation or paralyzation: Evidence for structural brain anomalies in body integrity identity disorder (BIID). PLoS One. 2016;11(11):e0165789. 27832097 10.1371/journal.pone.0165789 PMC5104450

[13] Hänggi J, Vitacco DA, Hilti LM, Luechinger R, Kraemer B, Brugger P. Structural and functional hyperconnectivity within the sensorimotor system in xenomelia. Brain Behav. 2017;7(3):e00657. 28293484 10.1002/brb3.657 PMC5346531

[14] Hilti LM, Hänggi J, Vitacco DA, et al The desire for healthy limb amputation: Structural brain correlates and clinical features of xenomelia. Brain. 2013;136(Pt 1):318–329. 23263196 10.1093/brain/aws316

[15] Hänggi J, Bellwald D, Brugger P. Shape alterations of basal ganglia and thalamus in xenomelia. Neuroimage Clin. 2016;11:760–769. 27330976 10.1016/j.nicl.2016.05.015 PMC4909827

[16] Saetta G, Hänggi J, Gandola M, et al Neural correlates of body integrity dysphoria. Curr Biol. 2020;30(11):2191–2195.e3. 32386532 10.1016/j.cub.2020.04.001

[17] Saetta G, Ruddy K, Zapparoli L, et al White matter abnormalities in the amputation variant of body integrity dysphoria. Cortex. 2022;151:272–280. 35462204 10.1016/j.cortex.2022.03.011

[18] Saetta G, Peter Y, Ruddy K, et al Feeling at home in a virtually amputated body: Neural and phenomenological effects of illusory embodiment of the desired body in body integrity dysphoria. J Psychiatr Res. 2025;184:395–404 40090220 10.1016/j.jpsychires.2025.02.055

[19] Berlingeri M, Devoto F, Gasparini F, et al Clustering the brain with “CluB”: A new toolbox for quantitative meta-analysis of neuroimaging data. Front Neurosci. 2019;13:1037. 31695593 10.3389/fnins.2019.01037 PMC6817507

[20] Gandola M, Zapparoli L, Saetta G, et al Brain abnormalities in individuals with a desire for a healthy limb amputation: Somatosensory, motoric or both? A task-based fMRI verdict. Brain Sci. 2021;11(9):1248. 34573269 10.3390/brainsci11091248 PMC8468102

[21] van Dijk MT, van Wingen GA, van Lammeren A, et al Neural basis of limb ownership in individuals with body integrity identity disorder. PLoS One. 2013;8(8):e72212. 23991064 10.1371/journal.pone.0072212 PMC3749113

[22] Bisiach E, Vallar G, Perani D, Papagno C, Berti A. Unawareness of disease following lesions of the right hemisphere: Anosognosia for hemiplegia and anosognosia for hemianopia. Neuropsychologia. 1986;24(4):471–482. 3774133 10.1016/0028-3932(86)90092-8

[23] Aoyama A, Krummenacher P, Palla A, Hilti LM, Brugger P. Impaired spatial-temporal integration of touch in xenomelia (body integrity identity disorder). Spatial Cogn Comput. 2012;12:96–110.

[24] Friston KJ, Ashburner J, Frith CD, Poline JB, Heather JD, Frackowiak RSJ. Spatial registration and normalization of images. Hum Brain Mapp. 1995;3(3):165–189.

[25] Ashburner J, Friston K. Nonlinear spatial normalization using basis functions. Hum Brain Mapp. 1999;7(4):254–266. 10408769 10.1002/(SICI)1097-0193(1999)7:4<254::AID-HBM4>3.0.CO;2-G PMC6873340

[26] Worsley KJ, Friston KJ. Analysis of fMRI time-series revisited—Again. NeuroImage. 1995;2:173–181. 9343600 10.1006/nimg.1995.1023

[27] Friston KJ, Stephan KE, Lund TE, Morcom A, Kiebel S. Mixed-effects and fMRI studies. NeuroImage. 2005;24(1):244–252. 15588616 10.1016/j.neuroimage.2004.08.055

[28] Flandin G, Friston KJ. Analysis of family-wise error rates in statistical parametric mapping using random field theory. Hum Brain Mapp. 2019;40(7):2052–2054. 29091338 10.1002/hbm.23839 PMC6585687

[29] Yarkoni T, Poldrack RA, Nichols TE, Van Essen DC, Wager TD. Large-scale automated synthesis of human functional neuroimaging data. Nat Methods. 2011;8(8):665–670. 21706013 10.1038/nmeth.1635 PMC3146590

[30] Serino A, Alsmith A, Costantini M, Mandrigin A, Tajadura-Jimenez A, Lopez C. Bodily ownership and self-location: Components of bodily self-consciousness. Conscious Cogn. 2013;22(4):1239–1252. 24025475 10.1016/j.concog.2013.08.013

[31] Vallar G, Ronchi R. Somatoparaphrenia: A body delusion. A review of the neuropsychological literature. Exp Brain Res. 2009;192(3):533–551. 18813916 10.1007/s00221-008-1562-y

[32] Gandola M, Invernizzi P, Sedda A, et al An anatomical account of somatoparaphrenia. Cortex. 2012;48(9):1165–1178. 21774922 10.1016/j.cortex.2011.06.012

[33] Saetta G, Michels L, Brugger P. Where in the brain is “the other's” hand? Mapping dysfunctional neural networks in somatoparaphrenia. Neuroscience. 2021;476:21–33. 34537314 10.1016/j.neuroscience.2021.09.007

[34] Loetscher T, Regard M, Brugger P. Misoplegia: A review of the literature and a case without hemiplegia. J Neurol Neurosurg Psychiatry. 2006;77(9):1099–1100. 16914766 10.1136/jnnp.2005.087163 PMC2077726

[35] So EL, Schauble BS. Ictal asomatognosia as a cause of epileptic falls: Simultaneous video, EMG, and invasive EEG. Neurology. 2004;63(11):2153–2154. 15596768 10.1212/01.wnl.0000145628.38030.3e

[36] Garbarini F, Fossataro C, Pia L, Berti A. What pathological embodiment/disembodiment tell us about body representations. Neuropsychologia. Dec 2020;149:107666. 33130159 10.1016/j.neuropsychologia.2020.107666

[37] Invernizzi P, Gandola M, Romano D, Zapparoli L, Bottini G, Paulesu E. What is mine? Behavioral and anatomical dissociations between somatoparaphrenia and anosognosia for hemiplegia. Behav Neurol. 2013;26(1–2):139–150. 22713395 10.3233/BEN-2012-110226 PMC5213125

[38] McGlone F, Wessberg J, Olausson H. Discriminative and affective touch: Sensing and feeling. Neuron. 2014;82(4):737–755. 24853935 10.1016/j.neuron.2014.05.001

[39] Legrain V, Damme SV, Eccleston C, Davis KD, Seminowicz DA, Crombez G. A neurocognitive model of attention to pain: Behavioral and neuroimaging evidence. Pain. 2009;144(3):230–232. 19376654 10.1016/j.pain.2009.03.020

[40] De Felice M, Ossipov MH. Cortical and subcortical modulation of pain. Pain Manag. 2016;6(2):111–120. 26984039 10.2217/pmt.15.63

[41] Wise SP . The primate premotor cortex fifty years after Fulton. Behav Brain Res. 1985;18(2):79–88. 3938285 10.1016/0166-4328(85)90064-6

[42] Passingham RE . Premotor cortex and preparation for movement. Exp Brain Res. 1988;70(3):590–596. 3384057 10.1007/BF00247607

[43] Shackman AJ, Salomons TV, Slagter HA, Fox AS, Winter JJ, Davidson RJ. The integration of negative affect, pain and cognitive control in the cingulate cortex. Nat Rev Neurosci. 2011;12(3):154–167. 21331082 10.1038/nrn2994 PMC3044650

[44] Moore JW, Middleton D, Haggard P, Fletcher PC. Exploring implicit and explicit aspects of sense of agency. Conscious Cogn. 2012;21(4):1748–1753. 23143153 10.1016/j.concog.2012.10.005 PMC3566545

[45] Perruchet P, Cleeremans A, Destrebecqz A. Dissociating the effects of automatic activation and explicit expectancy on reaction times in a simple associative learning task. J Exp Psychol Learn Mem Cogn. 2006;32(5):955–965. 16938039 10.1037/0278-7393.32.5.955

[46] Gu H, Salmeron BJ, Ross TJ, et al Mesocorticolimbic circuits are impaired in chronic cocaine users as demonstrated by resting-state functional connectivity. NeuroImage. 2010;53(2):593–601. 20603217 10.1016/j.neuroimage.2010.06.066 PMC2930044

