# Supplementary material for: The hidden side of body integrity dysphoria: aberrant limbic responses to dynamic touch
Source: Brain Commun. 2025 May 28;7(3):fcaf209. doi: 10.1093/braincomms/fcaf209 (PMC12150211; doi:10.1093/braincomms/fcaf209)
Supplement: fcaf209_Supplementary_Data [file fcaf209_supplementary_data.pdf]

Supplementary materials

The hidden side of body integrity dysphoria: aberrant limbic responses to dynamic touch

Laura Zapparoli[1,2*#], Eraldo Paulesu[1,2*#], Martina Gandola[3,4,5], Gerardo Salvato[3,4,5], Gianluca Saetta[6,7], Marika Mariano[1], Francantonio Devoto[1], Silvia Amaryllis Claudia Squarza[8], Mariangela Piano[8], Peter Brugger[9] & Gabriella Bottini[3,4,5]

[1] Psychology Department and NeuroMi – Milan Centre for Neuroscience, University of Milano-Bicocca, Milan, Italy

[2] fMRI Unit, IRCCS Orthopedic Institute Galeazzi, Milan, Italy

[3] Department of Brain and Behavioral Sciences, University of Pavia, Pavia, Italy

[4] Cognitive Neuropsychology Centre, ASST Grande Ospedale Metropolitano Niguarda, Milan, Italy

[5] NeuroMi, Milan Center for Neuroscience, Milan, Italy

[6] Professorship for Social Brain Sciences, Department of Humanities, Social and Political Sciences, ETH Zurich, Zurich, Switzerland

[7] Department of Adult Psychiatry, Psychiatry St. Gallen, Pfäfers, Switzerland

[8] Neuroradiology Department, ASST Grande Ospedale Metropolitano Niguarda, Milan, Italy

[9] Center for Psychiatric Research, Adult Psychiatry and Psychotherapy, University Hospital Zurich, Zurich, Switzerland

# Laura Zapparoli and Eraldo Paulesu contributed equally to this work

*** Corresponding authors**

Laura Zapparoli & Eraldo Paulesu
Psychology Department
University of Milano-Bicocca
Milan, Italy
Email: laura.zapparoli.unimib.it; eraldo.paulesu@unimib.it

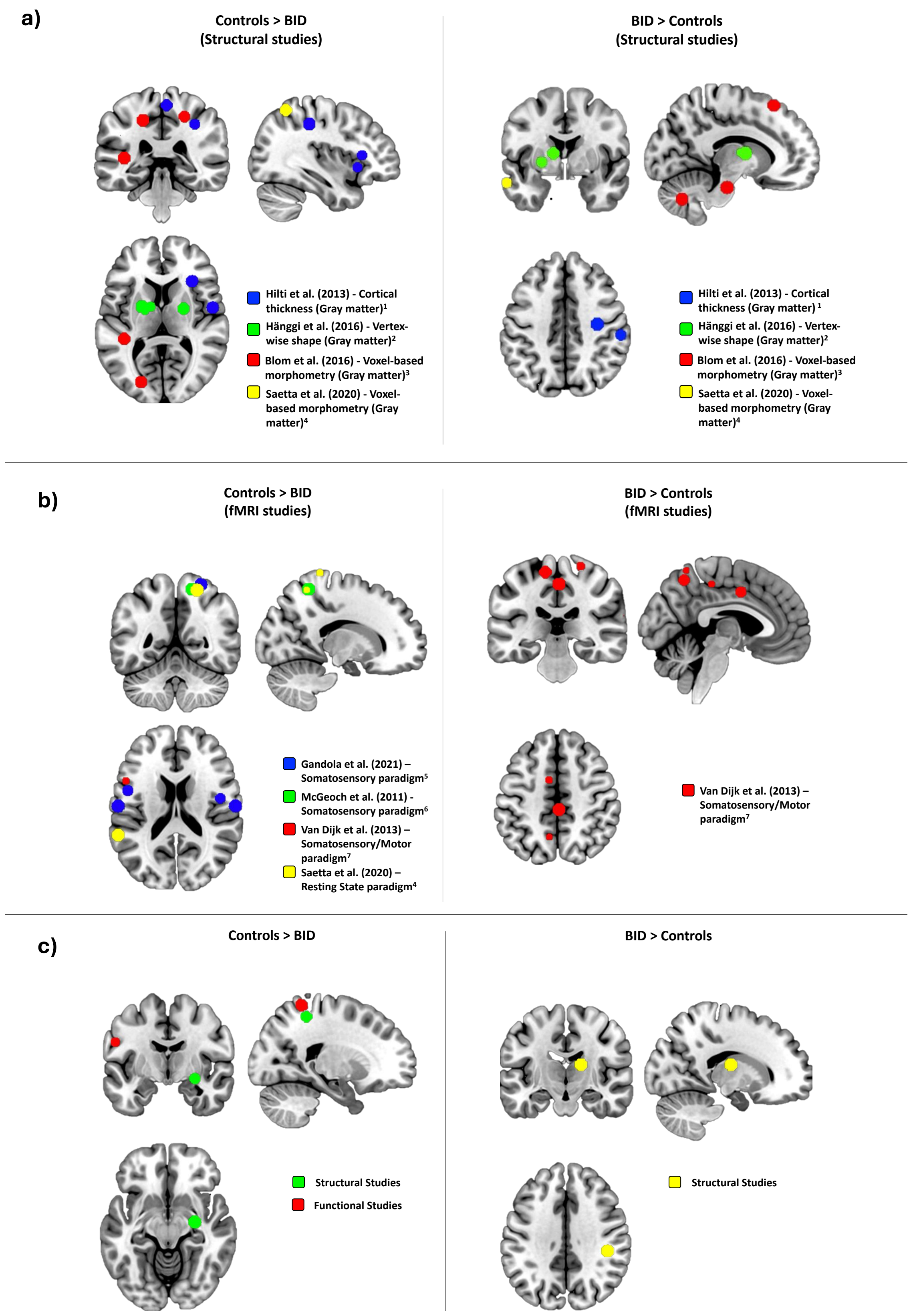

Figure S1. (a) Graphical illustration of the regional effects described in previous structural magnetic resonance imaging (MRI) studies. Images according to neurological convention (left is left). (b) Graphical illustration of the regional effects described in previous functional magnetic resonance imaging (fMRI studies). Images according to neurological convention (left is left). (c) Meta-analytical visualisation of the results of previous studies based on hierarchical clustering.

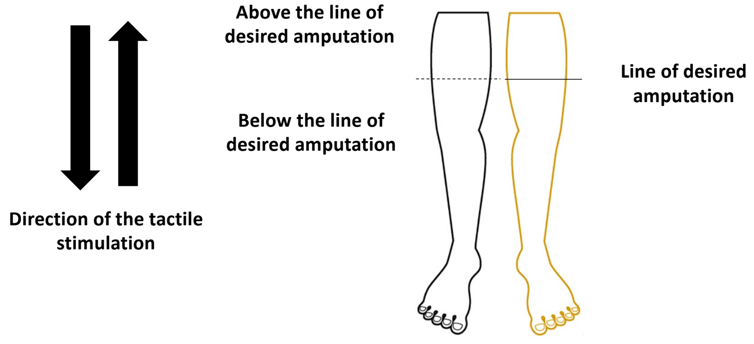

Figure S2. Graphical representation of the experimental paradigm.

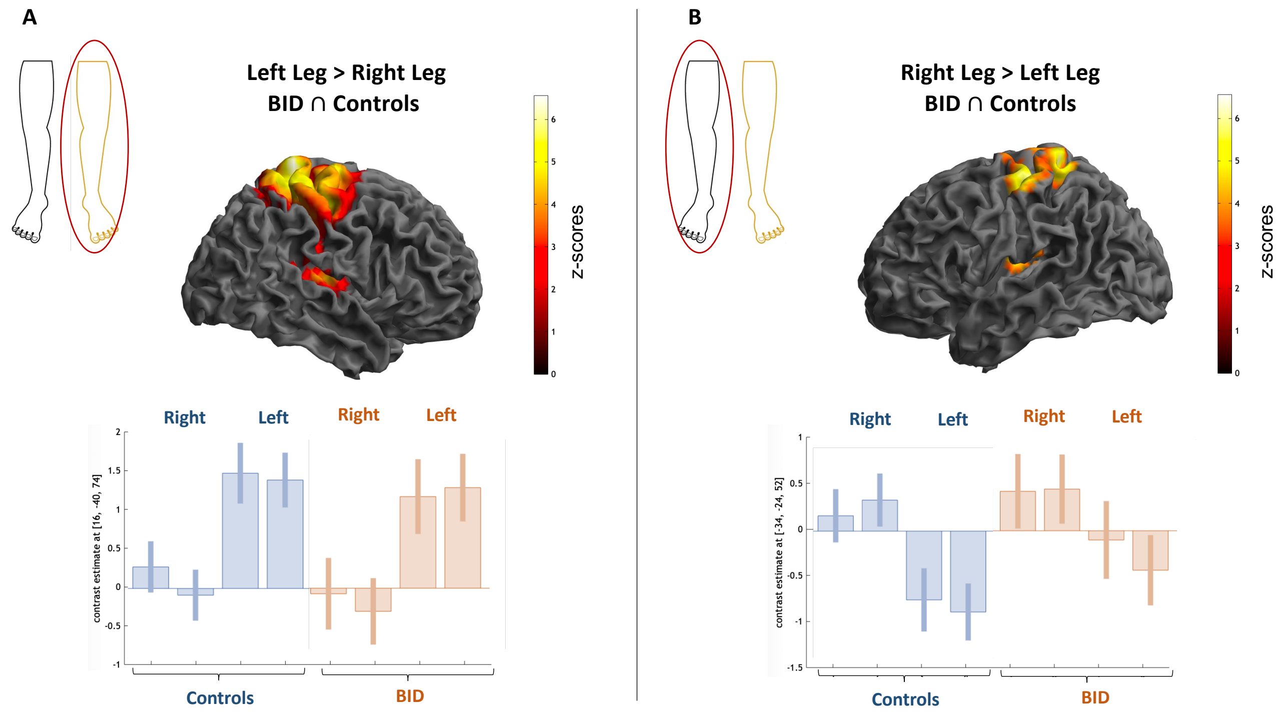

Figure S3. Full factorial ANOVA (N=23), conjunction analysis. Brain activations shared by the two groups for the tactile stimulation and plot of the hemodynamic response following the stimulation of (a) the left leg (postcentral gyrus: [16, -40, 74], Z-score: 5.88, $p<0.001$ at peak-level, $p<0.05$ Family Wise Error corrected at cluster level) and (b) the right leg (postcentral gyrus: [-34, -24, 52], Z-score > 8, $p<0.001$ at peak-level, p<05 Family Wise Error corrected at cluster level). Note that, in the BID group, the amputation desire targeted the *left* leg, without exception.

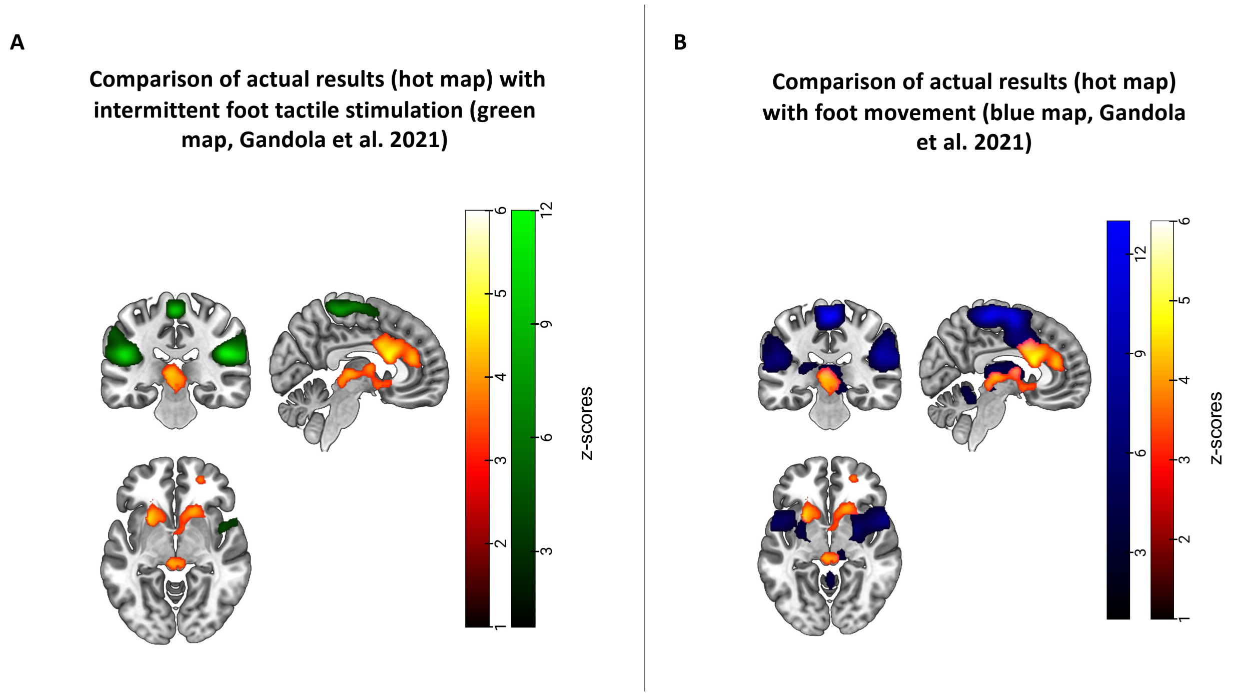

Figure S4. Overlap of our present findings (Full factorial ANOVA, N=23; p<0.001 at peak-level, p<05 Family Wise Error corrected at cluster level) with previous data (Gandola et al., 2021[5], Full factorial ANOVA, N=24; p<0.001 at peak-level, p<0.05 Family Wise Error corrected at cluster level), collected in the same sample of subjects, considering (a) intermittent foot tactile stimulation and (b) foot movement. This figure illustrates the pattern of hyperactivation recorded in this study and the main effect of foot sensory stimulation collected in our study in the same sample of individuals using a different tactile stimulation paradigm (Gandola et al., 2021)[5].

Table S1a. Metanalysis results: Controls > BID

Brain Region (BA)	Left Hemisphere				Right Hemisphere				Studies
	x	y	z	# Peaks	x	y	z	# Peaks	
Structural Studies									
Inferior frontal gyrus, opercular part (44)	-42	5	27	4	-	-	-	-	Blom et al., 2016 (2 peaks); Saetta et al., 2020 (2 peaks)
Postcentral Gyrus (3)	-	-	-	-	24	-43	54	5	Hilti et al., 2013 (3 peaks); Blom et al., 2016 (1 peak); Saetta et al., 2020 (1 peak)
Hippocampus (20)	-	-	-	-	28	-11	-11	4	Blom et al., 2016 (2 peaks); Hanggi et al., 2016 (2 peaks)
Functional Studies									
Precentral Gyrus (43)	-55	-3	27	4	-	-	-	-	van Dijk et al., 2013 (2 peaks); Gandola et al., 2021 (2 peaks)
Superior Parietal Lobule (5)	-	-	-	-	16	-47	66	3	McGeoch et al., 2011 (1 peak); Saetta et al., 2020 (2 peaks)

Table S1b. Metanalysis results: BID > Controls

Brain Region (BA)	Left Hemisphere				Right Hemisphere				Studies
	x	y	z	# Peaks	x	y	z	# Peaks	
Structural Studies									
Supramarginal Gyrus (3)					44	-31	35	3	Hilti et al., 2013 (2 peaks); Blom et al., 2016 (1 peak)
Thalamus	-	-	-	-	16	-14	15	3	Blom et al., 2016 (2 peaks); Hanggi et al., 2016 (1 peak)

Table S2a. Conjunction effect: Left Leg > Right Leg BID ∩ Controls. *FWER 0.05 correction (voxel level).

Brain Region (BA)	Left Hemisphere				Right Hemisphere			
	x	y	z	Z score	x	y	z	Z Score
Middle Cingulum (23)	-	-	-	-	10	-14	48	6.89*
Supplementary Motor Area (6)	-	-	-	-	14	-16	68	7.70*
Precentral Gyrus (4)	-	-	-	-	32	-20	58	7.70*
Postcentral Gyrus (3)	-	-	-	-	16	-40	74	> 8*
	-	-	-	-	30	-36	64	> 8*
Cerebellum 4-5	-12	-50	-18	5.01*	-	-	-	-
	-18	-48	-26	5.00*	-	-	-	-
	-16	-50	-22	4.96*	-	-	-	-
Insula	-	-	-	-	36	-18	10	7.32*
Putamen	-	-	-	-	30	-2	-6	3.62

Table S2b. Conjunction effect: Right Leg > Left Leg BID ∩ Controls. *FWER 0.05 correction (voxel level).

Brain Region (BA)	Left Hemisphere				Right Hemisphere			
	x	y	z	Z score	x	y	z	Z Score
Supplementary Motor Area (6)	-8	-10	48	3.61	-	-	-	-
	-8	-20	58	3.52	-	-	-	-
Precentral Gyrus (6)	-20	-12	76	4.04	-	-	-	-
	-16	-14	68	3.86	-	-	-	-
Paracentral Lobule (4)	-14	-26	80	3.93	-	-	-	-
	-12	-22	82	3.88	-	-	-	-
Postcentral Gyrus (3)	-34	-24	52	5.88*	-	-	-	-
	-22	-44	64	5.73*	-	-	-	-
	-18	-40	68	5.61*	-	-	-	-
	-16	-40	72	5.61*	-	-	-	-
	-48	-22	62	5.58*	-	-	-	-

Table S3. Correlations between BOLD response and Zurich Xenomelia Scale scores.

Bold Response at	Pure amputation desire*	Erotic attraction	Pretending behaviour
**[4,16,18]**	0.19*	0.54	0.27
**[-4,-22,2]**	0.3*	0.57	-0.2
**[4,-30,-16]**	0.50*	0.04	0.11
**[16,22,36]**	0.05*	0.43	0.18
**[56,6,42]**	-0.33*	0.18	0.19
**** Spearman's coeffcient***			

Supplementary references

1. Hilti LM, Hänggi J, Vitacco DA, et al. The desire for healthy limb amputation: structural brain correlates and clinical features of xenomelia. Brain. Jan 2013;136(Pt 1):318-29. doi:10.1093/brain/aws316
2. Hänggi J, Vitacco DA, Hilti LM, Luechinger R, Kraemer B, Brugger P. Structural and functional hyperconnectivity within the sensorimotor system in xenomelia. Brain Behav. Mar 2017;7(3):e00657. doi:10.1002/brb3.657
3. Blom RM, van Wingen GA, van der Wal SJ, et al. The Desire for Amputation or Paralyzation: Evidence for Structural Brain Anomalies in Body Integrity Identity Disorder (BIID). PLoS One. 2016;11(11):e0165789. doi:10.1371/journal.pone.0165789
4. Saetta G, Hänggi J, Gandola M, et al. Neural Correlates of Body Integrity Dysphoria. Curr Biol. 06 08 2020;30(11):2191-2195.e3. doi:10.1016/j.cub.2020.04.001
5. Gandola M, Zapparoli L, Saetta G, et al. Brain Abnormalities in Individuals with a Desire for a Healthy Limb Amputation: Somatosensory, Motoric or Both? A Task-Based fMRI Verdict. Brain Sci. Sep 21 2021;11(9)doi:10.3390/brainsci11091248
6. McGeoch PD, Brang D, Song T, Lee RR, Huang M, Ramachandran VS. Xenomelia: a new right parietal lobe syndrome. J Neurol Neurosurg Psychiatry. Dec 2011;82(12):1314-9. doi: 10.1136/jnnp-2011-300224
7. van Dijk MT, van Wingen GA, van Lammeren A, et al. Neural basis of limb ownership in individuals with body integrity identity disorder. PLoS One. 2013;8(8):e72212. doi:10.1371/journal.pone.0072212
